# Comparative Genomic Analysis of Warthog and Sus Scrofa Identifies Adaptive Genes Associated with African Swine Fever

**DOI:** 10.3390/biology12071001

**Published:** 2023-07-14

**Authors:** Wen Feng, Lei Zhou, Pengju Zhao, Heng Du, Chenguang Diao, Yu Zhang, Zhen Liu, Wenjiao Jin, Jian Yu, Jianlin Han, Edward Okoth, Raphael Mrode, Jian-Feng Liu

**Affiliations:** 1 National Engineering Laboratory for Animal Breeding, Key Laboratory of Animal Genetics, Breeding and Reproduction, Ministry of Agriculture; Frontiers Science Center for Molecular Design Breeding (MOE), College of Animal Science and Technology, China Agricultural University, Beijing 100193, China; wfeng@cau.edu.cn (W.F.); leiz@cau.edu.cn (L.Z.); zhaopengju2014@gmail.com (P.Z.); kimi-du@cau.edu.cn (H.D.); mghawk@163.com (C.D.); zhangyu040588@cau.edu.cn (Y.Z.); zhenliu@cau.edu.cn (Z.L.); jwjwenjiao@163.com (W.J.); cauyuj@cau.edu.cn (J.Y.); 2 Shenzhen Kingsino Technology Co., Ltd., Shenzhen 518107, China; 3 International Livestock Research Institute (ILRI), Nairobi 00100, Kenya; h.jianlin@cgiar.org (J.H.); e.okoth@cgiar.org (E.O.); r.mrode@cgiar.org (R.M.); 4 CAAS-ILRI Joint Laboratory on Livestock and Forage Genetic Resources, Institute of Animal Science, Chinese Academy of Agriculture Sciences (CAAS), Beijing 100193, China

**Keywords:** warthog, Kenyan domestic pig, genome assembly, African swine fever, lactate dehydrogenase B

## Abstract

**Simple Summary:**

This study aimed to elucidate the evolution of African Suidae and the molecular basis of their unique phenotypes, thereby attempting to fill these gaps. To this end, we performed the genome assembly and annotation of warthogs and Kenyan domestic pigs to identify the genetic resources responsible for warthog resistance against African swine fever. Our results showed that genes in the warthog genome relate to its adaptation to the African environment and that the gene lactate dehydrogenase B could be considered a tolerance gene to African swine fever. We believe that our study makes a significant contribution to the literature because our results provide valuable resources and data support for future evolutionary research on African pigs and genetic research adapted to unique local geographic environments in Africa. Additionally, the results of this study provide a new candidate gene for understanding the resistance mechanisms of warthogs against African swine fever.

**Abstract:**

Background: As warthogs (*Phacochoerus africanus*) have innate immunity against African swine fever (ASF), it is critical to understand the evolutionary novelty of warthogs to explain their specific ASF resistance. Methods: Here, we present two completed new genomes of one warthog and one Kenyan domestic pig as fundamental genomic references to elucidate the genetic mechanisms of ASF tolerance. Results: Multiple genomic variations, including gene losses, independent contraction, and the expansion of specific gene families, likely molded the warthog genome to adapt to the environment. Importantly, the analysis of the presence and absence of genomic sequences revealed that the DNA sequence of the warthog genome had an absence of the gene lactate dehydrogenase B (*LDHB*) on chromosome 2 compared with the reference genome. The overexpression and siRNA of *LDHB* inhibited the replication of the African swine fever virus. Combined with large-scale sequencing data from 42 pigs worldwide, the contraction and expansion of tripartite motif-containing (TRIM) gene families revealed that TRIM family genes in the warthog genome are potentially responsible for its tolerance to ASF. Conclusion: Our results will help improve the understanding of genetic resistance to ASF in pigs.

## 1. Introduction

Suidae is a family of artiodactyl mammals that originated 20–30 million years ago (mya) and consists of 15–17 extant species grouped into five genera [1]. As the most abundant and widely distributed member of the Suidae, the domestic pig (*Sus scrofa domesticus*) is a domesticated species of global importance as it constitutes a preferred source of animal protein for human consumption and serves as an important biomedical model. The common warthog (*Phacochoerus africanus*) is a wild member of the family Suidae that is naturally distributed in the grasslands, savannas, and woodlands of sub-Saharan Africa. The large, flat head of the warthog is covered with protective bumps and armed with four sharp tusks, presenting a significantly different appearance from that of domestic pigs.

Their unique geographical environment provides the Suidae with an African genetic basis in relation to their phenotypic traits, including pathogen challenges and fitness in resource-limited systems. Compared with the worldwide domestic swine herds, the common warthog (*Phacochoerus africanus*) presents natural immunity against African swine fever (ASF) and has no clinical symptoms following African swine fever virus infection [2].

ASF is a highly contagious, virulent disease in wild and domestic pigs, representing an acute, febrile, and hemorrhagic fever with a high rate of morbidity and mortality (up to 100%) [3]. ASF is classified by the World Organization for Animal Health as a List A disease, and has periodically and broadly savaged distinct countries and regions in Africa, Europe, South America, and Asia since its first report in Kenya in 1921, which killed pigs and devastated the swine industry severely [4]. Since 2018, it has spread to Eastern Asia and hit the majority of provinces in China, which is the world’s largest pig producer, raising concerns about global product supply and economic losses [5]. The financial loss from this ASF epidemic is estimated to be hundreds of billions of US dollars owing to mortality, culling, decreased reproduction, indirect economic loss, and a decrease in consumer surplus [6]. Additionally, ASF has already invaded islands in Southeast Asia, threatening 11 species of endemic pigs, including the Sulawesi warty pig (*Sus celebensis*), and causing a precipitous decline in wild pigs, such as the Bornean bearded pig (*Sus barbatus*) [7]. ASF is caused by a double-stranded DNA African swine fever virus (ASFV) with genomic lengths of 170–193 kb and 150–167 genes encoding more than 150 proteins, representing one of the most complex viruses discovered to date [8]. While live attenuated vaccines (LAVs) have shown promising results for against ASF, it is crucial to consider the potential risks associated with their use in non-endemic regions. The current LAV candidates for ASF are primarily based on the modifications of ASFV genotype II strains, with some involvement of genotype I strains. The purpose of these modifications is to weaken the virus’s virulence while still allowing it to induce an immune response in vaccinated animals. To address these concerns, the thorough testing and monitoring of LAVs are necessary before their deployment [9].

Genomic comparison between asymptomatic warthogs and swine helps to elucidate the genetic mechanisms of host resistance to ASFV infection and adaptation to African contexts. Using the assembled genomes of the common warthog (*Phacochoerus africanus*), red river hog (*Potamochoerus porcus*), and East Asian Diannan small-ear pig (*Sus scrofa*), the previous report by Xie et al. provided new insights into the evolutionary history and divergent genetic adaptations of the African Suidae [10]. 

In the present study, we generated two de novo genome assemblies of African Suidae with one warthog and one Kenyan domestic pig, which enhanced our understanding of the evolution of African Suidae and enabled us to unravel the molecular basis of their unique phenotypes. The fully annotated genomes allowed us to identify the genetic content of the presence/absence variation (PAV) in African Suidae and provided new insights into the evolution of the Suidae genome. We present the speciation history of African pigs, and the orthologous genes in Suidae were used to reveal the genetic changes in the warthog genome responsible for their adaptation to the African environment, including gene family expansions and contractions. These results identified new candidate genes for understanding the molecular mechanisms underlying warthog resistance to ASF.

## 2. Materials and Methods

### 2.1. Sample Preparation for Genome Assembly

We assembled the warthog and Kenyan domestic pig genomes in order to understand the evolution of African Suidae. The study utilized tissues from a warthog that naturally deceased in Nairobi National Park, Kenya, including the heart, spleen, kidneys, and lungs. The tissues of Kenyan domestic pigs, including the kidney, heart, liver, spleen, lung, tonsil, gastro-hepatic lymph node (GHLN), mesenteric lymph node (MSLN), and submandibular lymph node (SMLN) were collected from six Kenyan domestic pigs in Homa Bay, Kenya. DNA and RNA were isolated from the tissues and used for sequencing. DNA isolated from the livers was used for genome assembly, and RNAs from all the tissues mentioned above were used for genome annotation (Table S1). 

### 2.2. Sample Preparation for Demographic Histories

Fourteen common warthogs and seven Kenyan domestic pigs were sampled from three localities around Kenya, and six pig ears were collected from Meishan (Table S2). These individuals were genetically unrelated.

Animal experiments and related sample collection and treatments were approved by the Institutional Animal Care and Use Committee (IACUC) of the International Livestock Research Institute (ILRI) (ref no. IACUC-RC2018-17). All procedures were performed in accordance with the ILRI IACUC protocol 11.

### 2.3. DNA and RNA Extraction and Sequencing

Genomic DNA was extracted from liver of warthogs and Kenyan domestic pigs for genome assembly using the MagAttract HMW DNA Kit (Qiagen, Germantown, MD, USA) for 10× genomics according to the manufacturer’s protocol. A 10× Chromium library was prepared according to the manufacturer’s instructions (Chromium™ Genome Library Kit & Gel Bead Kit v2) and sequenced on an Illumina HiSeq2500 sequencing system (Illumina, CA, USA) with a 2 × 250 bp read metric.

Genomic DNA for demographic histories was extracted from tissues or peripheral blood mononuclear cells (PBMCs) using DNeasy Blood & Tissue Kits (Qiagen) according to established protocols and checked for quality and quantity using NanoDrop2000 (Thermo Fisher Scientific, Waltham, MA, USA) and agarose gel electrophoresis. A total DNA amount of 1 μg or higher was used for DNA sequencing. The total DNA was sequenced using an Illumina HiSeq 2500.

Total RNA was isolated from the tissues according to the standard TRIzol method protocol (Invitrogen, Carlsbad, CA, USA). RNA degradation and contamination were monitored using 1% agarose gel electrophoresis. The concentration of total RNA was measured using a Qubit RNA Assay Kit on a Qubit 2.0 Fluorometer (Life Technologies, Carlsbad, CA, USA). RNA samples that met the criteria of having an RNA integrity number (RIN) value of 7.0 or higher and a total RNA amount of 5 μg or higher were included and batched for RNA sequencing. RNA sequencing libraries were constructed using the Kapa RiboErase (Roche, Basel, Switzerland), with 3 μg of rRNA-depleted RNA, according to the manufacturer’s recommendations. Libraries were sequenced using the Illumina NovaSeq 6000 S4 platform according to the manufacturer’s instructions, with a minimum data size per sample of 5 G clean reads (corresponding to 150 bp paired-end reads). 

Raw sequenced DNA-Seq and RNA-Seq data were obtained from the NCBI Sequence Read Archive (BioProject number PRJNA691462).

### 2.4. Reads Alignment to the Reference Genome Sus Scrofa 11.1

The RNA-Seq raw data were trimmed based on quality control for downstream analyses using the following steps. First, BBmap (Version v0.38) automatically detected the adapter sequence of reads and removed those reads containing Illumina adapters [11]. The Q20, Q30, and GC contents of the clean data were calculated using FastQC for quality control and filtering [12]. The resulting sequences were mapped to the reference genome (*Sus scrofa 11.1*) using HISAT2 [13]. The NCBI *Sus scrofa11.1* annotation was used as the transcript model reference for alignment, as well as for all protein-coding genes and isoform expression-level quantifications. Finally, feature counts (from subread v2.0.1) were used to calculate the number of read counts [14]. 

### 2.5. Genome Assembly

The genome was assembled using the default parameters of the Supernova assembler (v2) designed by Illumina 10× Genomics. The scaffolds were connected using a pseudogap to obtain a genomic draft. Fixed gaps were added between the two scaffolds, and a sealer [15] and the Gapcloser [16,17] were used to fill the gaps.

### 2.6. Evaluation of Genome Assembly

To evaluate genome quality using *Sus Scrofa 11.1* as reference, raw reads were mapped to the warthog and Kenyan domestic pig genomes using BWA v0.7.17 [18]. Next, the coding gene sequences and transcripts of *Sus Scrofa 11.1* were mapped to the assembled genomes, and genome completeness was verified by 4104 benchmarking universal single-copy orthologs to the genome using BUSCO v.3.0.2b [19]. Finally, the two assembled genomes were mapped to *Sus Scrofa 11.1* using MUMer v3.23 [20]. The parameter was used with “-maxmatch -1 100–c500”.

### 2.7. Read Alignment and Variant Calling of DNA Sequence Reads

To facilitate better read mapping, the three criteria of quality control (QC) were implemented by FastQC with “−q 20 −−thread=6 −−length_required=120 −−n_base_limit = 6.” Filtered reads from all individuals were aligned to the *Sus scrofa 11.1* reference genome using the Burrows–Wheeler Aligner (BWA v0.7.17) [18]. We performed duplicate marking, base quality recalibration, duplicated read removal, and mapping statistics (i.e., depth coverage) using Picard, GATK, and SAMtools. Finally, the alignment files (BAM) were used for subsequent analyses.

The BAM files of 42 pigs were used for single nucleotide variant (SNV) detection at the population scale using GATK (v4.1.4). The GATK command was run with the functions “HaplotypeCaller”, “GenomicsDBImport”, “GenotypeGVCFs”, “MergeVcfs”, and “SelectVariants” to generate genotype calls in the variant call format (VCF). Moreover, the GATK command was run with parameters “QD < 2.0, QUAL < 30.0, FS > 200.0 ReadPosRankSum < −20.0” to filter each SNV VCF file. The dbSNP database (ftp://ftp.ncbi.nih.gov/snp/organisms/archive/pig_9823/VCF/, accessed on 16 August 2017) was used to identify the novel genetic variations. The gene structure annotation of SNPs was performed using ANNOVAR [21]. The reference genome was *Sus scrofa 11.1* and the annotation file was downloaded from UCSC (https://hgdownload.soe.ucsc.edu/goldenPath/susScr11, accessed on 20 August 2020).

### 2.8. Annotation of Assembled Genomes

The genome annotation included two main sections: structural and functional. Structural annotation included repetitive sequence identification, noncoding gene prediction, and coding gene prediction.

The repetitive sequence consisted of three parts: (1) de novo prediction: repetitive sequences of the whole genome were predicted using RepeatModeler (2.0.1) [22]; (2) the repetitive sequences were mapped to the UniProt database using BLAST [23] to remove sequences with lengths greater than 50 bp; and (3) data-driven homology annotation: the repetitive sequences obtained in the previous two steps were merged into RepBase (v20181026) [22] and then RepeatMasker (v4.1.1) [24] was used to perform soft annotation at the whole genome level.

Three procedures pertained to non-coding and coding genes: (1) de novo prediction: AUGUSTUS (v3.4.0) [25] and GeneMark-ES (v4.62) [26] were used for genome prediction; (2) data-driven homology annotation: HISAT2 (v2) [27] was used to perform quality control of RNA-seq data, StringTie [28] was used to assemble the transcripts, and TransDecoder [29] was used to predict the open reading frame (ORF) structure; and (3) combining the prediction results: the results of the first two steps were filtered and merged to obtain the final annotation results using EVidenceModeler [30].

Functional annotation consisted of two steps: (1) the predicted proteins were aligned with the UniProt database using DIAMOND [31] and (2) Pfam domain annotation was performed using PfamScan [32,33] based on the Pfam database.

### 2.9. Annotation of Merged Genomes

The *Sus Scrofa 11.1* genome was merged with the unique sequences of the warthog and 14 additional domestic pig genomes. Repetitive sequence prediction was ignored because of short sequences.

### 2.10. Non-Coding Genes and Coding Gene Prediction

The *Sus scrofa* genome of OrthoDB 10.1 (https://www.orthodb.org/, accessed on 8 January 2019) [34] was used as the protein dataset for homology comparison, and BRAKER2.1.5 [35] software was used for gene structure prediction. In the BRAKER2 process, GeneMark-ES [26] was used to perform de novo genome analysis to obtain seed genes. Seed genes were then mapped to the protein dataset using DIAMOND [31] with fine mapping of the splicing sites using Spaln v2.3.3d [36]. Using the mapping information, high-quality genes were obtained using GeneMark-EP+ [37]. Finally, the high-quality genes were used as the training set and the AUGUSTUS (v3.4.0) [25] parameters were trained to obtain the final structure prediction result.

### 2.11. Function Annotation

Blastx [23] was used to map the obtained protein sequences to a non-redundant protein sequence database (value = 1 × 10^−6^, minimum percentage of identical matches = 70%). InterProScan (v5.48-83.0) [32] was used to annotate the protein domains and GO terms.

### 2.12. The Identification of Presence–Absence Variation (PAV)

The assembled genomes, including those of warthogs and Kenyan domestic pigs, and data from 14 additional genomes, including those of Landrace, Largewhite, Pietrain, ½ Landrace-¼ Duroc-¼ Yorkshire, Berkshire, Hampshire, Wuzhishan, Göttingen, Bama, Jinhua, Meishan, Tibetan, Bamei, and Rongchang pigs, downloaded from public databases (Table S11), were mapped to the Duroc genome (*Sus Scrofa 11.1*) using Blastn [23] to identify the presence–absence variation in each genome. 

### 2.13. Phylogeny Construction and Estimate of Divergence Time

We constructed a phylogenetic tree of warthogs, Kenyan pigs, Duroc pigs, cattle, mice, and humans using maximum likelihood analysis of a concatenated alignment of 3,489 single-copy orthologous genes shared with their genomes [PhyML] [38]. iTOL was used to reconstruct and optimize the phylogenetic tree. The divergence time between the warthogs and Kenyan and Duroc pigs was estimated using the Markov Chain Monte Carlo (MCMC) tree program, as implemented in the phylogenetic analysis of the maximum likelihood (PAML) package [39]. Calibration times (89.3, 96.8, and 62.0 mya) were derived from the TimeTree database.

### 2.14. Gene Family Expansion and Contraction

We determined the expansion and contraction of the gene ortholog clusters by comparing the cluster size differences between the ancestor and each of the current warthog, Kenyan, Duroc, cattle, mouse, and human clusters (*p* < 0.05) using OrthoFinder [40] and the CAFE program [41], which is based on a probabilistic graphical model.

### 2.15. Phylogenetic Analysis

The distance between genomes was determined using the Minhash method [42]. First, genome sketches were conducted using different genome sequences to complete the calculation of different genome distance matrices and construct evolutionary trees. Finally, the constructed evolutionary tree was visualized using iTOL [43].

The demographic history of warthogs; Kenyan domestic pigs; Duroc, Landrace, and Meishan pigs; and Chinese wild boars was analyzed using Pairwise Sequentially Markovian Coalescent (PSMC) v.0.6.5 software [44]. The parameter generation interval (g) was 5, and the mutation rate of each generation was set to 1.25 × 10^−8^.

### 2.16. Selection Signature Analysis

The composite likelihood ratio (CLR) was used to detect the selection signal in the warthog population using SweepFinder [45] with a continuous window of 100 kb of the genome.

### 2.17. Gene Functional Analysis

The functional KEGG pathway [46] enrichment of genes using selection signature was clustered using KOBAS3.0 (http://kobas.cbi.pku.edu.cn/kobas3/genelist/, accessed on 2 July 2021). The pathways with q < 0.05 were considered significant. We confirmed that only the KEGG pathway map images were used for our Supplementary Data. According to the Kanehisa Laboratories policy, no permission is required when KEGG pathway map images are used only in Supplementary Data.

### 2.18. The Expression of Fluorescent Proteins in 3D4/21 Cells after Plasmid Transfection

To validate the resistance of lactate dehydrogenase B *(LDHB)* to ASF, fluorescent protein expression in porcine alveolar macrophage 3D4/21 cells transfected with pEGFP-C1-LDHB and siRNA-LDHB were detected. The 3D4/21 porcine alveolar macrophages used in this study were purchased from the American Type Culture Collection (ATCC CRL-2843). The experiments were conducted at the Institute of Military Veterinary Medicine, Academy of Military Medical Science. The genotype II ASFV strain SY18 (GenBank accession number MH766894) used in this study was supplied by the Institute of Military Veterinary Medicine, Academy of Military Medical Science. To establish a connection between protein expression and virus replication, the SFV-SY18-∆CD2v/UK mutant contains EGFP replacing the CD2v gene, enabling the visualization of the virus using a fluorescent microscope (Nikon TE2000-U, Tokyo, Japan). The detection protocols described by Borca et al. were implemented in order to observe the fluorescence associated with virus presence [47,48]. The culture and viral titer determination of ASFV SY18 have been described in a previous study [24]. 

TsingKe Biological Technology (Beijing, China) constructed the pEGFP-C1-LDHB, siRNA-LDHB, blank control pEGFP-C1, and blank control siRNA vectors. The forward and reverse sequences of siRNA-LDHB are 5′-GCAAGGUUUCGCUAUCUUATTUAAGAUAGCGAAACCUUGCTT—3′ and 5′-GCAAGGUUUCGCUAUCUUATTUAAGAUAGCGAAACCUUGCTT—3′. Primary alveolar 3D4/21 macrophages were seeded in 6-well plates at a concentration of 2 × 10^6^/mL and incubated at 37 °C with 5% CO_2_ in an RPMI 1640 maintenance medium. When the cell density was approximately 70–90%, the pEGFP-C1-LDHB, siRNA-LDHB, pEGFP-C1, and siRNA vectors were transfected into the cells according to the Lipofectamine™ 3000 (Thermo, MA, USA) and incubated at 37 °C with 5% CO_2_ for 24 h. At this point, the cell fusion efficiency reached 100%. 

In the study, the Median Tissue Culture Infectious Dose (TCID50) of ASFV-SY18-∆CD2v/UK is 106.5/mL. Based on the equation Plaque Forming Units (PFU) = cell counts × Multiplicity of Infection (MOI) = 0.7 × TCID50, each well of cells was exposed to a known titer of ASFV at an MOI of 5. After a 2 h incubation with the virus, the culture medium was replaced with 1640 medium containing 2% FBS. The cells were then infected with the ASFV-SY18-∆CD2v/UK, digested, and harvested via centrifugation at 48 h post-infection. The cells were observed under a confocal laser scanning fluorescence microscope (Olympus LSCMFV500).

## 3. Results

### 3.1. De Novo Assembly of Warthog and the Kenyan Domestic Pig Genomes

To present comprehensive genomic resources for the Suidae in Africa, we generated two newly assembled genomes, one for common warthog and another for Kenyan domestic pig, using the 10× Genomics “Linked-Read” sequencing technology. We generated 1.20 billion and 1.17 billion 150-nt chromium-linked paired-end reads, which resulted in a genome coverage depth of approximately 69× (180.32 Gb) and 68× (176.97 Gb) for warthog and Kenyan domestic pig, respectively (Table 1).

The warthog and Kenyan domestic pig genomes were assembled and scaffolded using a Supernova assembler with a diploid pseudo-haplotype style, and the resulting scaffolds were gap-filled using the Sealer application [15]. After removing the contaminations from adaptors and viral genomes, the final assemblies yielded 19,366 scaffolds with a total length of 2.417 Gb and an N50 of 13.75 Mb and 13,380 scaffolds with a total length of 2.445 Gb and N50 30.52 Mb for the warthog and Kenyan domestic pig genomes, respectively (Table 2).

The quality of the genome assemblies was evaluated (Table S3) using the following steps. First, we mapped all chromium-linked paired-end reads to our genome assemblies with no large structural variation and an average sequence identity of 99.32%, indicating that our assemblies were correct and contained most of the information in the raw reads. The further assessment of genome completeness revealed that our genome assemblies recovered 91.5% and 93.0% of the 4104 single-copy orthologs in mammalian gene groups from the warthog and Kenyan domestic pig, respectively, which were comparable to the Benchmarking Universal Single-Copy Orthologs (BUSCO) completeness score of 93.0% for the reference genome built from a Duroc pig (*Sus scrofa 11.1*). We observed that 89.6% and 96.26% of the 41,909 transcripts from the pig reference genome could be mapped to the warthog and Kenyan domestic pig assemblies (≥90% query coverage), respectively. Altogether, these results indicate the high quality of the de novo assembled genomes of warthogs and Kenyan domestic pigs.

### 3.2. Annotation of the Assembled Genomes

Using 227,840 orthologous protein isoforms (*Sus scrofa*) from OrthoDB 10.1 (https://www.orthodb.org/, accessed on 8 January 2019) as gene evidence, we employed the BRAKER2 pipeline to predict protein-coding genes (PCGs) by integrating the ab initio and homology-based approaches.

As a result, 17,781 and 22,127 PCGs were identified in the warthog (with an average gene length of 21,907 bp and 6.7 exons per gene) and Kenyan domestic pig (21,929 bp and 6.5 exons) assemblies, of which 59.3% and 52.4%, respectively, were homologous with the 27,445 PCGs annotated in the pig reference sequence. We found 1668 novel PCGs (Table S4) in the warthog assembly, which were supported by mRNA transcripts and had an ortholog in the non-redundant databases (NR) of NCBI, InterPro, or UniportDB (Swiss-Prot and TrEMBL). Notably, these unique warthog PCGs contained the largest percentage of immune-related genes as compared with pig PCGs and were significantly enriched in KEGG pathways related to “Pathways in cancer” and “Metabolic pathways” (adjusted *p*-value < 0.01), which could explain why the warthogs have very strong genetic resistance/tolerance to a variety of endemic parasitic and viral diseases.

### 3.3. The Expansion and Contraction of Gene Family Evolution

**Figure 1:**
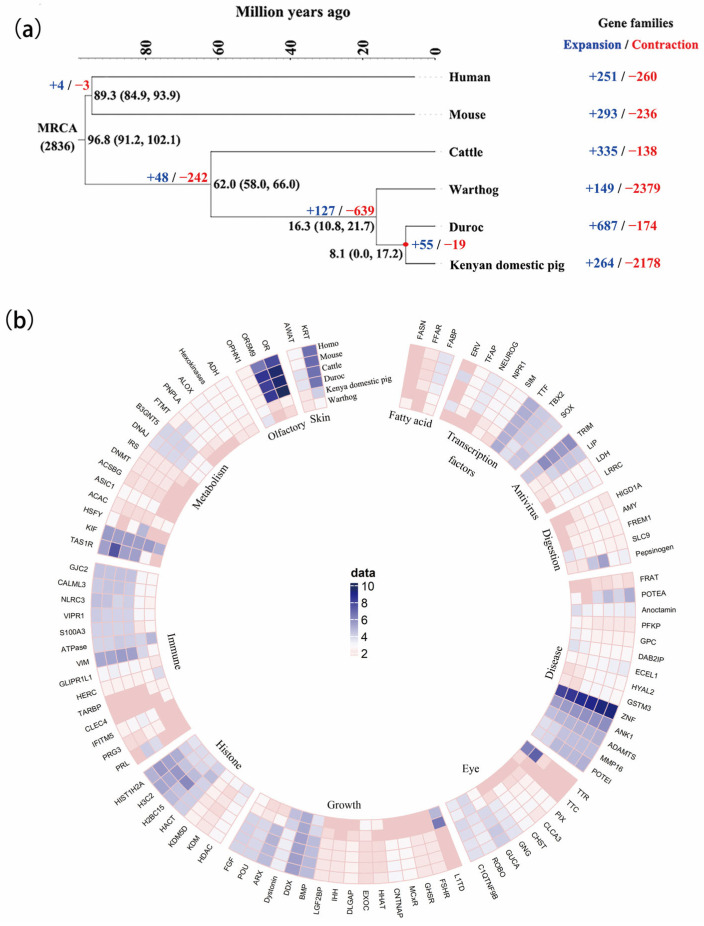
The expansion and contraction of gene families. (**a**) The phylogenetic tree was constructed with 423 orthologs from six mammals using OrthoMCL with a Markov cluster algorithm. Divergence time was estimated using the approximate likelihood calculation method in conjunction with a molecular clock model. A bar within a branch indicates the 95% confidence interval of divergent time. The positive and negative numbers adjacent to the taxon names are gene family numbers of expansion/contraction obtained from the CAFE analysis. (**b**) The gene families whose total number of family members varies between warthog and other species. Data represent the log10 of total number of genes in each gene family. Each slide is a gene family, and each sector is a gene function cluster.

To tease apart the gene family patterns of expansion and contraction between the warthog and Kenyan domestic pigs, we performed a computational analysis of gene family evolution (CAFE) using orthologous gene families in the human (GCF_000001405.40), mouse (GCF_000001635.27), cattle (GCF_002263795.2), warthog, Duroc (GCF_000003025.6), and Kenyan domestic pig (Figure 1a and Tables S5 and S6). A *t*-test for gene numbers in gene families comparing warthogs with other species detected 67 significant gene families (Table S7). We identified 67 gene families involved in immunity, facial function, digestion, metabolism, and skin (Figure 1b). Genes belonging to *TRIM*, *LDH*, *LIP*, *SLC9*, *HERC*, *CLEC4*, *TARBP*, and *PRL* families participate in the immune and antiviral responses [49,50]. Our results showed that the number of contracted gene families was greater than that of expanded gene families in warthogs. Kenyan domestic pigs show similar gene family expansion and contraction patterns. Specifically, 2379 contracted and 149 expanded gene families were observed in warthogs, whereas 2178 contracted and 264 expanded gene families were observed in Kenyan domestic pigs. We also observed that a substantial number of gene families were unique to warthog or Kenyan domestic pigs. Among these expanded gene families, 104 were unique to warthogs. Massive expansion of phospholipid-transporting ATPases, previously noted in the pig reference genome, has functional implications for muscle electrical conductivity [51] and thermotolerance [52]. The expanded gene *NFKB1* identified in the warthog plays a crucial role in the NF-KB pathway [53]. Among the 37 expanded gene families shared by the warthog and Kenyan domestic pig, genes *MX1*, *MX2*, *ETS1*, and *PSPC1* are involved in the immune response [54,55,56]. 

### 3.4. Phylogenic Analysis and Demographic History

**Figure 2:**
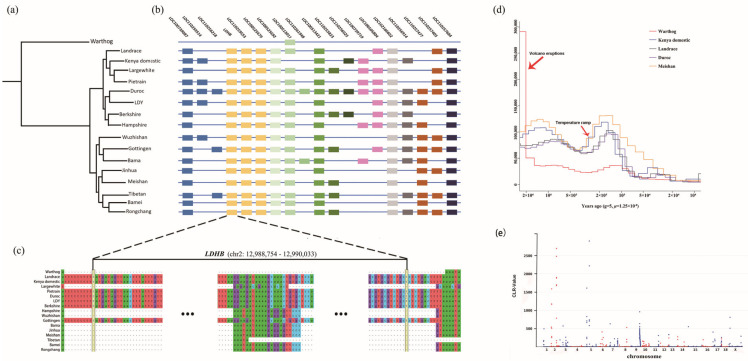
The Demographic history and selective genes in adaptive evolution of African Suidae. (**a**) Demographic history of pigs using 17 assembled genomes. The genomes of warthogs and Kenyan domestic pigs were assembled in this study, while 15 additional genomes were downloaded from public databases. (**b**) The distribution of PAVs that affect the coding regions of 19 genes with known annotations. The same color indicates that these genes are located on the same chromosome. (**c**) Coding sequence alignment of gene LDHB in chromosome 2 of the 17 assembled genomes. (**d**) Demographic history of pigs. Generation time (g) = 5; transversion mutation rate (μ) = 1.25 × 10^−8^. (**e**) Plot of CLR values in the warthog.

To further explore the relationship between warthogs and domestic pigs, we aligned the two assembled genomes with other 15 domestic pig genomes that are publicly available (Table 3). Genetic distances between the genomes, evaluated using MinHash, are shown in Figure 2a. The phylogenetic tree clearly separates warthogs and domesticated pigs into two main branches, which implies a divergent genetic background. The long branch length between the warthog and domestic pigs indicates a long evolutionary history between the genera *Phacochoerus* and *Sus*, in comparison with the domestication of *Sus scrofa*. Within the domestic branches, pig breeds seem to be clustered by their geographic regions, European and Asian. Considering the unique phenotype and living environments, we expect Kenyan domestic pigs to belong to an independent phylogenetic branch. Contrary to our expectations, we found the Kenyan domestic pig clustered together with European pigs, which implies they are either genetically close to each other or derived from a common ancestor. Intriguingly, we found that the Gottingen pig clustered together with Asian pigs rather than with European pigs, even though it geographically belongs to European pigs. This implies that the genetic contribution of Asian pigs to the artificial breeding process of Gottingen pigs is more significant than that of European pigs.

To better understand the evolutionary history of warthogs and Kenyan domestic pigs, a pairwise sequentially Markovian coalescent (PSMC) was employed to estimate their demographic histories and effective population sizes (Ne) (Figure 2d). Given that the phylogenetic tree separated domestic pigs into two clusters, we also used two representative pig breeds in the PSMC analysis: Duroc/Landrace for European pigs and Meishan pigs for Asian pigs. Between 2 mya and one mya, the number of warthogs was sharply reduced. Approximately 2 Mya, a large number of volcanoes continued to erupt in Kenya and caused severe and long droughts in the region [57], which may have been one of the reasons for the decline in warthog populations. Approximately 200,000 years ago, the global temperature experienced drastic fluctuations with rapid changes from cold to warm, which led to a significant increase in the number of wild pig species worldwide [58]. The current study showed that approximately 3000 years ago, the effective population sizes of European and African domestic pigs tended to be highly similar.

### 3.5. Signature Analysis of Selection

To test the genomic signal formed through the positive selection of warthogs in the natural environment, a selective sweep of the warthog genome was performed using CLR. Regions with CLR-values >100 (top 0.2%) were considered strongly selected (Figure 2e). In total, 138 important windows were screened. Chromosomes 2 and 5 were subjected to strong selection. A total of 186 genes were identified in these windows, of which 43 genes had detailed annotation information, including *MID2* and many interleukin family genes, such as *IL19, IL1A, IL20*, and *IL24*. The protein encoded by *MID2* is a member of the tripartite motif (TRIM) family, which is involved in paracrine TNFα signaling [59]. KEGG pathway analysis was performed on these genes, and 15 significant pathways were detected [46] (Q < 0.05, Table S8). These pathways are associated with viral infections, immunity, skin diseases, and the retina.

### 3.6. Massive Presence-Absence Sequences among the Suidae Genomes

Intraspecific presence/absence variants (PAVs) are important sources of genetic diversity and divergence that contribute to an organism’s ability to adapt to a specific habitat. To capture the set of PAVs in all available accessions of Suidae, we performed a multi-genome alignment of 16 genomes to the pig reference genome and retained the unaligned scaffolds and structural variations (insertions or deletions longer than 50 bp) from the aligned scaffolds as PAVs. As expected, compared with all pig breeds, the warthog had the highest number of PAVs (165,452), ranging from 31,939 in 13.3 Mb to 98,767 in 27.8 Mb (Table S9). The further exploration of the genomic distribution revealed that the density of PAVs decreased from the distal region(s) toward the centromere within a chromosome and showed significant enrichment in the intergenic regions (chi-square test, *p* < 0.05).

By comparing the distribution patterns of PAVs and protein-coding regions along the genome, we found 67 protein-coding regions linked to PAVs in the reference pig genome, 19 of which had known annotations (Table S10). The distribution of the PAVs linked to these 19 genes in different species and breeds is shown in Figure 2b. Interestingly, *LDHB* was mapped to two distinct genomic locations in the pig reference genome: chromosome 5 (chr5:51810932–51831218) and chromosome 2 (chr2:12988754–12990033). The entire LDHB sequence mapped to chromosome 2 of *Sus scrofa 11.1* was missing from the warthog (Figure 2c), whereas the copy of *LDHB* on chromosome 5 was present in the warthog genome.

**Figure 3:**
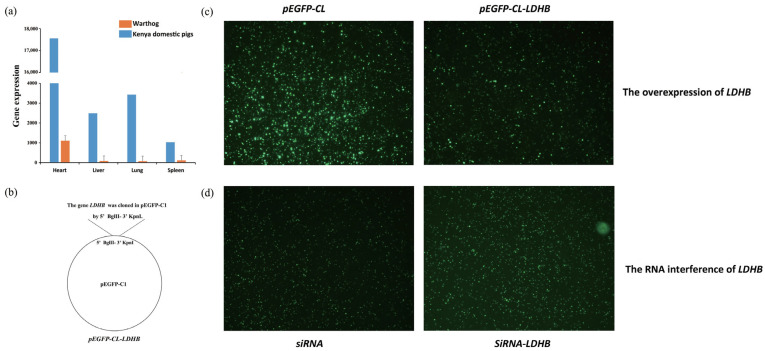
Validation of the effect of *LDHB* against ASFV. (**a**) Gene expression of *LDHB* in the heart, liver, lung, and spleen of warthogs and Kenyan domestic pigs. (**b**) The gene *LDHB* has been cloned into pEGFP-C1 by BglII-KpnI. (**c**) The expression of fluorescent proteins in plasmid-transfected 3D4/21 cells after infection with ASFV SY18 strain when *LDHB* was overexpressed. (**d**) The expression of fluorescent proteins in plasmid-transfected 3D4/21 cells after being infected with ASFV SY18 strain after interfering with the RNA of *LDHB*. The stronger the fluorescence intensity, the greater the amount of ASFV protein.

The expression levels of *LDHB* in the heart, lungs, liver, and spleen of warthogs were compared with those in Kenyan domestic pigs. The expression level of the gene *LDHB* in these four tissues was more than 10 times higher in warthogs than in Kenyan domestic pigs (Figure 3a). 

To determine the replication efficiency of ASFV treated with LDHB overexpression and siRNA in gene *LDHB*, 3D4/21 cells transfected with either pEGFP-Cl-LDHB or the siRNA-LDHB vector were infected with the ASFV SY18 strain (MOI = 5). The gene *LDHB* was cloned into pEGFP-C1 using BglII-KpnI (Figure 3b), which represents the overexpression of *LDHB*. After 48 h of infection, the fluorescence intensity and proportion of 3D4/21 cells transfected with pEGFP-Cl-LDHB were reduced compared with those transfected with the pEGFP-Cl (blank control) vector (Figure 3c), indicating that ASFV replication was decreased when LDHB was overexpressed. In contrast, the fluorescence intensity and proportion of 3D4/21 cells transfected with siRNA-LDHB were higher than those of the blank control (Figure 3d). The fluorescence intensity reflects the inhibitory effect of *LDHB* on ASFV replication.

## 4. Discussion

Our study used 10× genomic sequencing technology for long-read genomic DNA sequencing of warthogs and Kenyan domestic pigs, which are highly adapted to the natural environment in Africa. The size of our warthog genome is 36 Mb smaller than the common warthog genome reported by Xie et al. and the length is 0.08 Mb [10]. The quality of our genome was slightly weaker than that of the previous study but comprised a lower amount, and the quality of our genome was good enough to perform the following analysis. The tissue used for DNA extraction was isolated from a naturally deceased warthog, and the lack of immediate tissue preservation may be the primary factor contributing to DNA and RNA degradation. In contrast, the tissue used to assemble the Kenyan domestic pig genome came from a laboratory source. Both the DNA and RNA quality of the Kenyan domestic pig are higher than that of the warthog. Furthermore, the Kenyan domestic pig belongs to the domestic species and is genetically closer to the reference genome genes. Therefore, the quality of genome assembly and genome annotation in the Kenyan domestic pig is higher than that in the warthog. These results provide valuable resources and data support for future evolutionary research on African pigs and genetic research on adaptation to unique local geographic environments.

Our PSMC results showed that the population size (Ne) of warthogs declined during the Last Glacial Period (115,000–11,700 years ago), contrary to the results of Xie et al. [10]. Our results showed that the population sizes of all Suidae declined because of the cold climate [60]. We also noted that the Ne of Kenyan domestic pigs was close to that of Meishan pigs 105 years ago, and then close to that of European pigs. Many studies have found that Kenyan pigs in East Africa not only have European ancestors but also a high frequency of alleles from the Far East [61,62]. Approximately 3000 years ago, there was a human resurgence in Africa. The populations of Kenya, Tanzania, and Ethiopia flooded with people from Eurasia [63]. Perhaps it was due to this population that the genes of European pigs infiltrated African pigs. Later, in the 17th century, with the Indian Ocean trade, genes of southern Chinese pigs infiltrated African domestic pigs. The gradual colonization of Africa in modern times and the strengthening of European commercial pig breeds have resulted in the dominant genes of European pigs being gradually fixed in East Africa. Some genes and breeds of native African domestic pigs have almost completely disappeared. 

The CAFE analyses of Suidae genomes in this study provide new insights into the genetic basis of local adaptations to diverse environments on the African continent. Our analyses suggest that common warthogs, which are well-adapted to savannah conditions, may have evolved adaptive signals in several aspects of sensory perception, including immunity, digestion, and metabolism (Figure 1b). Combined with our gene family evolution and signature analyses, the TRIM family genes were expanded in the Duroc and contracted in the Kenyan domestic pig and warthog. TRIM proteins, which stands for tripartite motif-containing proteins, play a crucial role in the immune response to pathogens. They are induced by both type I and type II interferons, which are important signaling molecules involved in the immune response [64]. Type I interferons are produced in response to viral infections and play a key role in activating the immune system to fight off the infection. Type II interferons, on the other hand, are mainly produced by immune cells and are involved in regulating immune responses against both viral and bacterial infections [65]. Several studies have shown that TRIM family genes are involved in the negative regulation of viral transcription [66] and positive regulation of I-κB kinase/NF-κB signaling [67]. Previous studies have reported that proteins recovered by ASFV can inhibit the activation of NF-κB [68]. Overall, TRIM proteins play a crucial role in the immune response to pathogens and are important for the host defense against infections. Further research is ongoing to understand the specific functions and mechanisms of different TRIM proteins in the immune system. TRIM family genes may be one of the reasons why warthogs and Kenyan domestic pigs show resistance to ASFV [69,70].

By comparing the genome structure of warthogs to the genomes of domestic pigs, we found that warthogs completely lacked the gene *LDHB* on chromosome 2. *LDHB* has been shown to play an important role in classical swine fever infection, and its overexpression can reduce the replication of classical swine fever virus [71]. *LDHB*, an enzyme involved in the conversion of lactate to pyruvate, has been found to play a crucial role in the energy metabolism of tumor cells. In addition to its role in tumor metabolism, *LDHB* has also been implicated in immune cell function. Studies have shown that the overexpression of *LDHB* in T cells enhances their respiration and cytokine production when exposed to lactic acid, a byproduct of anaerobic glycolysis that is commonly elevated in the tumor microenvironment [72,73]. The expression levels of *LDHB* in the heart, lungs, liver, and spleen of warthogs were compared with those in Kenyan domestic pigs. The expression level of the gene *LDHB* in the four tissues was more than 10 times higher in warthogs than in Kenyan domestic pigs (Figure 3a). To verify whether *LDHB* has an inhibitory effect on ASFV, the expression of fluorescent proteins in 3D4/21 cells transfected with plasmids after infection with ASFV SY18 (Figure 3c,d) was observed. The overexpression and RNA interference of *LDHB* demonstrated its inhibitory effect on ASFV replication. 

## 5. Conclusions

In conclusion, we assembled the genomes of warthogs and Kenyan domestic pigs and explored the evolution and gene family changes in African and other pigs. Considering the transcriptomic data from warthogs and Kenyan domestic pigs, we demonstrated that LDHB was able to inhibit the replication of ASFV. These newly discovered molecular genetic markers of ASF resistance will help improve ASF resistance in different pig breeds.

## Figures and Tables

**Table 1:** Summary of raw sequencing data.

	The Number of Raw Base (bp)	The Number of Raw Reads	Coverage Depth	Read Length
Warthog	180,321,392,700	1,202,142,618	69×	150 bp
Kenya domestic pig	176,972,667,300	1,179,817,782	68×	150 bp

**Table 2:** Statistic of two assembled genomes.

	Warthog	Kenya Domestic
Assembled genome size (Gb)	2.417	2.445
number of ‘N’ (Mb)	40.3	21.8
N content of whole genome (%)	1.64	0.883
Contig N50 (kb)	132.54	144.01
Number of scaffold	19,366	13,380
Total scaffolds (>=1 Mb)	275	138
Total scaffolds (>=100 kb)	493	262
Total scaffolds (>=10 kb)	2479	2253
Total scaffolds (>=1 kb)	20,477	14,837
Scaffold N50 (Mb)	13.75	30.52
Scaffold N75 (Mb)	6.1	15.47
Scaffold N90 (Mb)	1.6	5.24
Average scaffold length (kb)	120.017	166.2
Longest scaffold (Mb)	65.64	100

**Table 3:** Assembled genomes used in the study.

Breeds	Project NO.	Breeds	Project NO.
Warthog	PRJNA691462	Duroc (Sus scrofa11.1)	PRJNA13421
Kenya domestic	PRJNA691462	½ Landrace- ¼ Duroc- ¼ Yorkshire (LDY)	PRJNA392765
Bama	PRJNA478804	Ellegaard Gottingen minipig	PRJNA176189
Wuzhishan	PRJNA144099	Tibetan	PRJNA186497
Jinhua	PRJNA309108	Hampshire	PRJNA309108
Bamei	PRJNA309108	Landrace	PRJNA309108
Meishan	PRJNA309108	LargeWhite	PRJNA309108
Pietrain	PRJNA309108	Berkshire	PRJNA309108
Rongchang	PRJNA309108		

## Data Availability

The raw whole 10× genomics sequencing data of warthog and Kenyan domestic pig, the RNA-Seq raw data of 28 samples, and the DNA-seq raw data of 42 samples are all available in the NCBI Sequences Read Archive (BioProject number: PRJNA691462).

## Supplementary Materials

The following supporting information can be downloaded at: https://www.mdpi.com/article/10.3390/biology12071001/s1, Table S1: Summaries of RNA sequencing reads; Table S2: Statistic of whole genome sequencing of 25 pig breeds; Table S3: Quality assessments of assembled genomes; Table S4: Specific and annotated PCGs of Warthog comparing to reference genome; Table S5: The contracted gene family observed; Table S6: The expanded gene family observed; Table S7: The gene families whose total number of family members varies between warthog and other species; Table S8: Analysis of KEGG enrichment of strong selective genes in warthog; Table S9: The number and length of PAV sequences; Table S10: Protein coding gene in PAVs of *Sus scrofa 11.1*. References [74,75,76,77,78,79,80,81] are cited in Supplementary Materials.
