# Supplementary material for: Comparative Genomic Analysis of Warthog and Sus Scrofa Identifies Adaptive Genes Associated with African Swine Fever
Source: Biology (Basel). 2023 Jul 14;12(7):1001. doi: 10.3390/biology12071001 (PMC10376286; doi:10.3390/biology12071001)
Supplement: Supplementary file 1 [file biology-12-01001-s001.zip › biology-2441164-supplementary/Supplementary.pdf]

Genomes Comparisons Provide Insights into Adaptive Genes for African Swine Fever

Genomic TE composition

Identifying repetitive sequences can effectively improve the accuracy of genome annotation. Transposable elements (TEs), one of the main and ubiquitous genomic repetitive elements (**Table S11**), are the driving forces in shaping genomic architecture and evolution. In total, 4,039,334 TEs occupied 42.82% (1,053 Mb) of the entire warthog genome, two-thirds of them were assigned to a specific family. In total, 4,247,450 TEs occupied 42.94% (1,061 Mb) of the entire Kenyan domestic pig genome. As observed in previous studies [1, 2], the most abundant TEs were retrotransposons (accounted for ~95% of TEs). Both LINEs and SINES together accounted for 82% and 81%, while DNA transposons took only 5.5% and 5.7% of the warthog and Kenyan domestic pig genomes (Figure S1**a**). LINEs accounted for more than half of TEs, thereby being the most abundant pig TEs in the both two assembled genomes, which were contradictory to the early observation that more quantity of SINEs (66.8%)than LINEs (14.1%) in Chinese and European pigs [3]. Family L1/CIN4 had major contributions, accounting for 93.3% and 92.6% of classified LINEs in the warthog and Kenyan domestic pig genomes, respectively. Then Kimura distance-based copy divergence analysis and correction with the CpG content of each TE were performed using RepeatMasker. The divergence pattern indicated the most ancient SINEs at 20 mya and one burst of LINEs at 60 mya, but no recent expansion of TEs in the two genomes. The warthog and Kenyan domestic pig genomes shared a similar pattern expansion and contraction of TEs (Figure S1**b**).

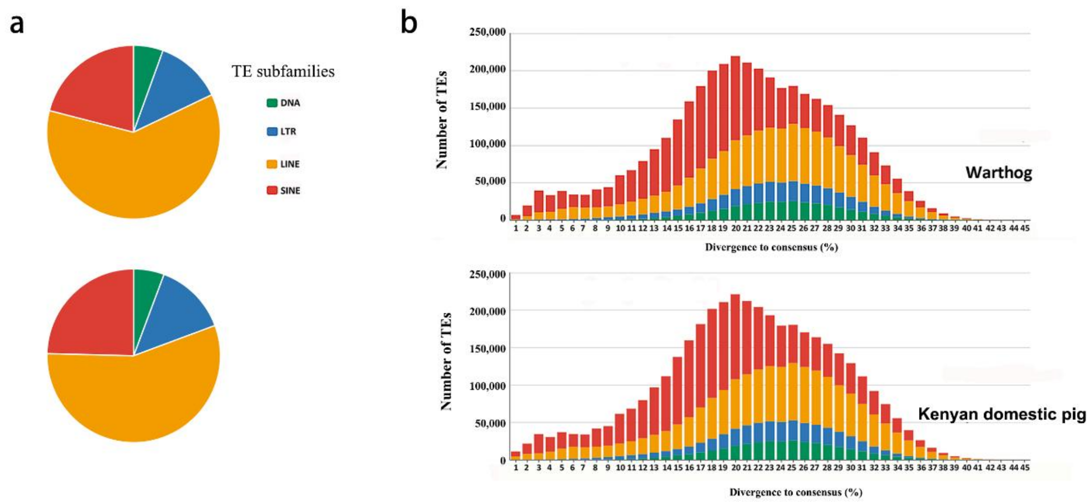

Figure S1. Genomic structure of the warthog and Kenyan domestic pig genomes and gene prediction. (**a** Proportions of DNA transposons, LTR, LINE, and SINE retrotransposons in the two assembled genomes. (**b** History of TE accumulation in the warthog and Kenyan domestic pig genomes. The stacking plots were used to show the divergence distribution either by summing all four TE classes or separately for each family.

Phylogenetic analysis

First, a phylogenetic tree was constructed with all 123 pigs (Figure S**2**). A total of warthogs were used as the outgroup. The result showed that Kenyan and Nigerian domestic pigs belonged to the same branch of European pigs. However, Kenyan domestic pigs were separated from European pigs before the Nigerian ones. The evolutionary direction of the Chinese pig breeds in this study was essentially the same as that obtained in previous research, where the northern domestic pigs were separated first, followed by pigs in the central and finally the southern Chinese pig

breeds.

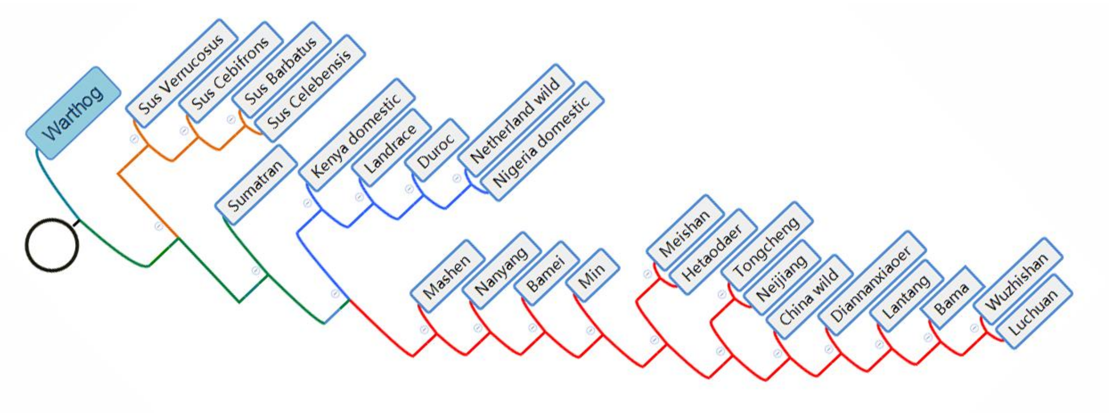

Figure S2. Neighbor-joining tree constructed from SNPs using MEGA X.

Next, principal component analysis (PCA) was performed using the above identified genome-wide SNPs (Figure S3). Similar with the phylogenetic relationship, the Kenyan and Nigerian domestic pigs were closely related to European pigs, though they can still be distinguished from Landrace and Duroc pigs.

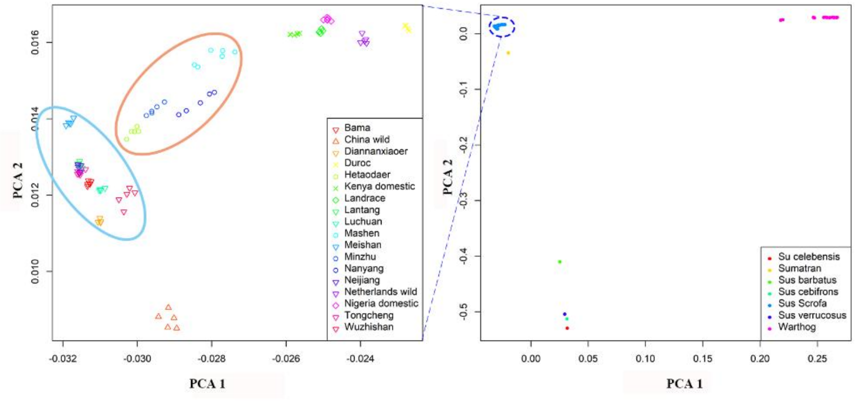

Figure S3. PCA analysis using the SNPs of 25 pig breeds. The right one used all 25 populations and the left one used the *Sus scrofa* pigs. Pigs in the dark blue circle were from Europe and Africa, those in the red circle were from North China, and those in the light blue circle were from South China. The triangles in the middle bottom were

wild pigs from South China.

An admixture analysis was further performed for these based on the genetic information of the population. The ancestors of the 25 pig breeds were artificially divided into K from 2 to 10, and the pedigree composition of different pig populations was analyzed (Figure S**4**). When K=6, the warthog was completely separated from the other *Sus* species. At the same time, there was genetic exchange between northern Chinese pigs and European pigs. When K ranged from 2 to 7, Kenyan domestic pigs carried genetic information from southern Chinese pigs. This was consistent with the results of previous studies that showed that East African domestic pigs had high-frequency alleles from the Far East [4]. Mashen pigs located in Shanxi carried genetic information of European and northern Chinese pigs when K ranged from 2 to 6. More interestingly, when K was 7, Mashen pigs were distinguished from other pig populations. When K was 9, Nigerian domestic pigs and the Netherlands wild boars were distinguished from other European pig populations. When K increased from 2 to 10, Kenyan domestic pigs shared the same ancestor as Duroc and Landrace pigs.

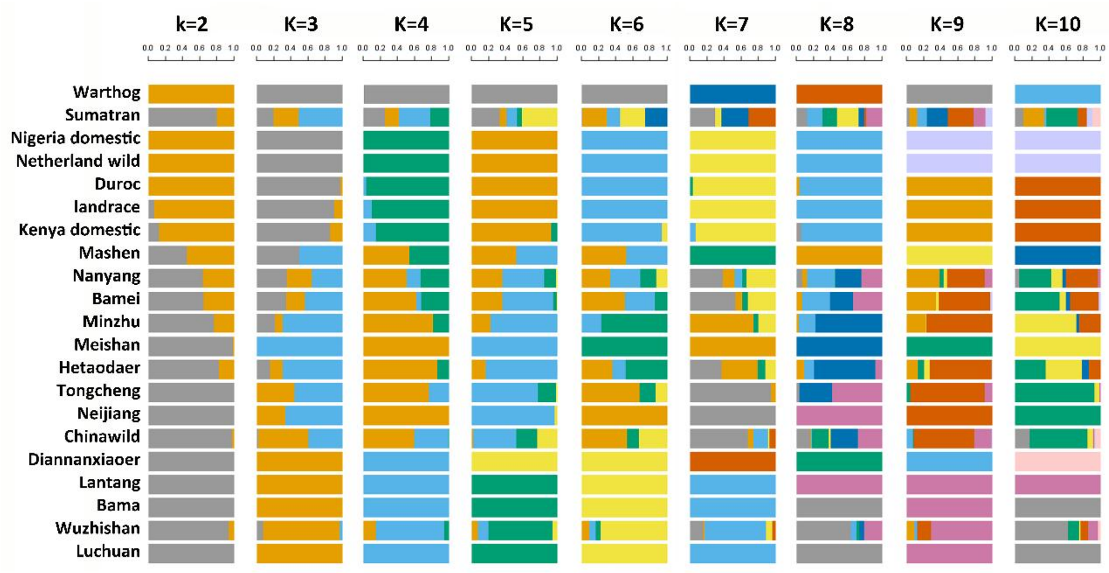

Figure S4. Structure analysis from k = 2 to 10 using ADMIXTURE.

Selection signature analysis

Further to understand the different characteristics of Kenya domestic pig comparing with Asian and European domestic pigs, the Pi and XP-EHH between Kenyan domestic and Duroc (Figure S**5a and b**), Kenyan domestic and Landrace (Figure S**5c and d**), and Kenyan domestic and Southern Chinese domestic pigs (Figure S**5e and 5f** were analyzed. The signature results showed that chromosomes 3 and 15 were strongly selected using different algorithms. A total of 195 genes were identified from the top 5% of the regions that were strongly selected **Table S11**). Among these genes, *CASP10*, *CASP8*, and *CFLAR*, located on chromosome 15, were involved in the immune and disease pathways. In the results of Kenya domestic - Duroc (**Table S12**) and Kenyan domestic – Landrace (**Table S13**), gene TRIM3 were strongly selective.

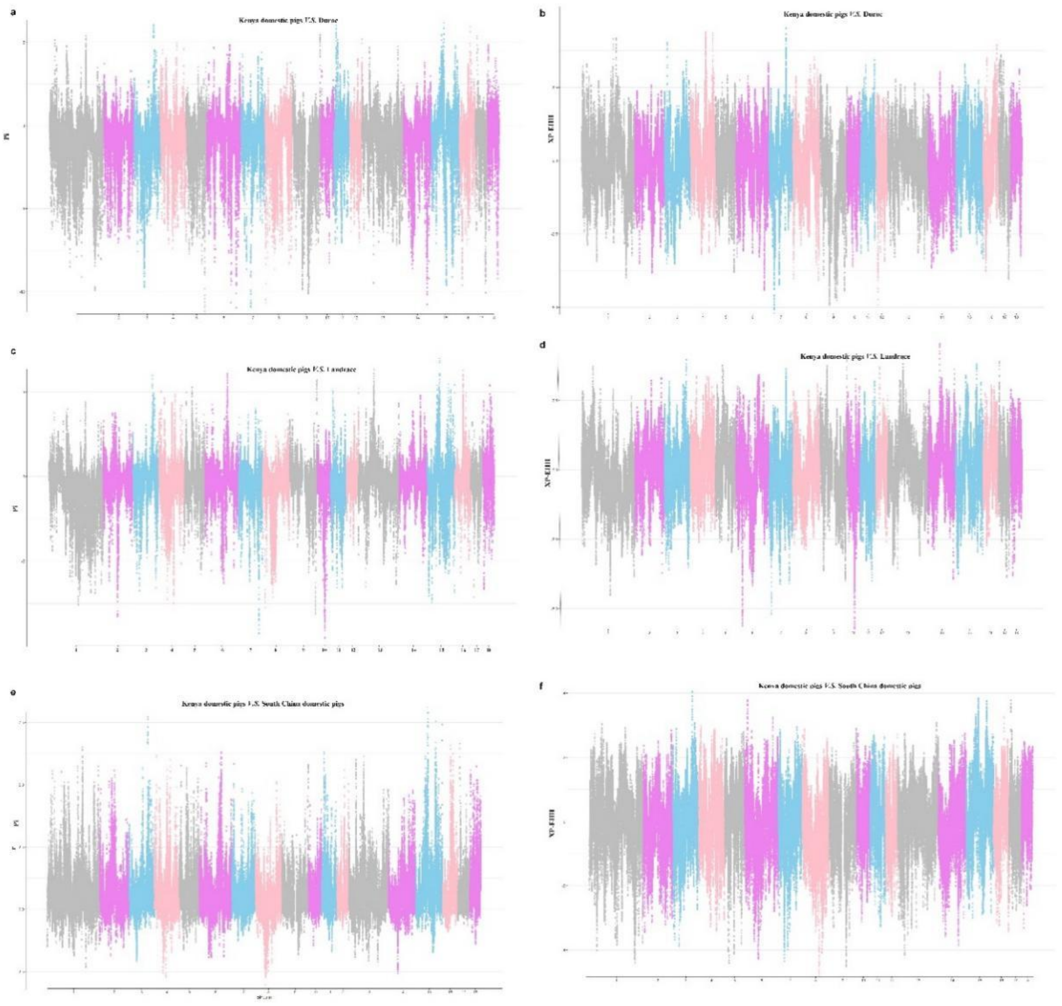

Figure S**5.** Selection signature analysis in populations. a. Plot of Pi in Kenyan domestic pigs and Duroc pigs. b. Plot of XP-EHH in Kenyan domestic pigs and Duroc pigs. c. Plot of Pi in Kenyan domestic pigs and Landrace pigs. d. Plot of XP-EHH in Kenyan domestic pigs and Landrace pigs. e. Plot of Pi in Kenyan domestic pigs and Southern Chinese domestic pigs. f. Plot of XP-EHH in Kenyan domestic pigs and Southern Chinese domestic pigs.

Methods

Phylogenetic analysis

A phylogenetic tree was constructed to understand the genetic relationship between

Kenyan domestic pigs and other domestic pigs, and the genetic evolutionary relationship between warthogs and Kenyan domestic pigs. First, the VCF file was converted into a PLINK format file using PLINK, and then the PLINK parameter "--distance" -matrix" was used to calculate the IBS matrix between individuals. The phylogenetic tree was visualized using MEGA-X [5].

Principal component analysis (PCA) was performed using GCTA [6] and PLINK with the parameter "--grm --pca 3." The population structure was analyzed using ADMIXTURE [7] with K=2 to K=10 for genetic clustering, and the convergence standard was 0.01. Then, ggot2 of the R package was used to visualize the PCA and population structure results of the obtained eigenvector values.

Selection signature analysis

The Pi and XP-HEE between Kenyan domestic pigs and Duroc pigs, Kenyan domestic pigs and Landrace pigs, and Kenyan domestic pigs and Southern Chinese domestic pigs (Bama, Diannanxiaoer, Luchuan, Lantang, and Wuzhishan) were analyzed with an overlapping continuous window of 100 kb and a step size of 10 kb.

Supplementary materials 2

SNP distribution of warthog and Kenyan domestic pigs

SNPs and InDels were detected among the warthog and Kenyan domestic pig genomes compared to the pig reference genome *Sus Scrofa* 11.1. A total of 36,840,924 SNPs and 5,534,671 InDels were present in the warthog while 6,533,170 SNPs and 2,021,513 InDels were observed in Kenyan domestic pig. Further comparisons with the pig SNP database (GCA_000003025.6, the European Bioinformatics Institute, EBI) showed that 75% and 33% of the SNPs identified in the warthog and Kenyan domestic pig were novel, respectively (Figure S**6a**). The higher numbers of both novel and total SNPs in the warthog than in Kenyan domestic pig were attributed to their significantly different genetic divergence and/or differentiation from the Duroc pig.

After the annotation of these SNPs, we found that the distribution of SNPs located in different gene regions shared the same pattern by the warthog and Kenyan domestic pig (Figure S**6b**). The intergenic regions contained most of the SNPs at 52.7% in the warthog and 54.0% in Kenyan domestic pig, followed by introns, 3' and 5' untranslated regions (UTRs), exon regions, and upstream and downstream regions. A comparison showed that the number of SNPs in coding genes, whether in their introns or exons, was greater than that in lncRNAs. The number of SNPs in the 3'-TUR of both coding genes and lncRNAs was higher than that in the 5'-UTR, similar with early findings in Meishan pig and human genomes [8, 9]. Specifically, in the

exon regions, 227,253 and 39,942 functional SNPs were present in the warthog and Kenyan domestic pig, respectively; out of these, 81,311 and 14,683 were non-synonymous, 665 and 130 stop-gain, and 138 and 43 stop-loss SNPs, respectively, while the rest were synonymous (Figure S6**c**).

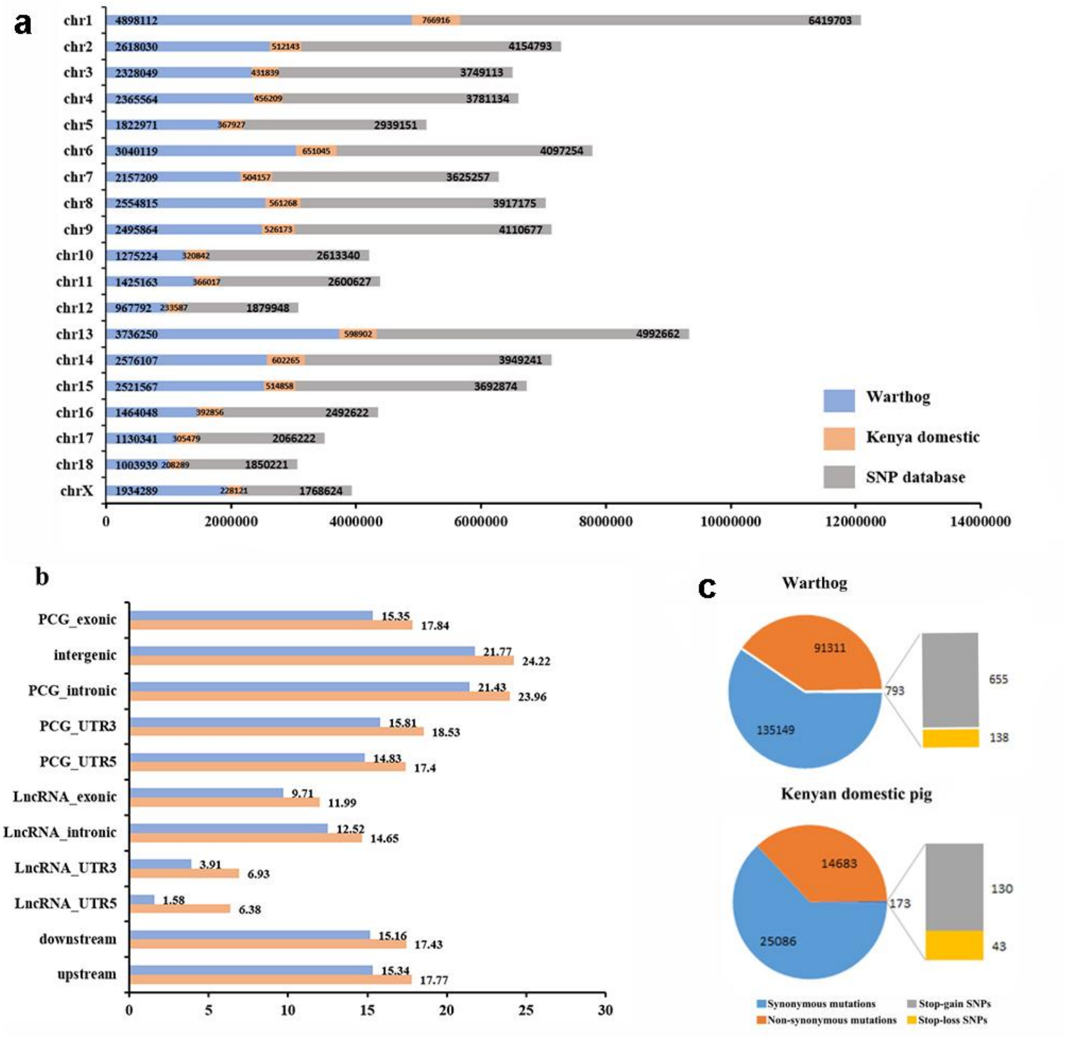

Figure S**6** SNP distribution of warthogs and Kenyan domestic pigs and estimation of effective population size (Ne) using resequencing data. a. Nucleotide diversities of warthogs, Kenyan domestic pigs, and the SNP database. b. Gene annotation of SNPs. Bar plots represent the $\log_2$ of the number of SNPs in various functional regions. c. The distribution of SNP in the exon.

Reference

1. Santos AA, Penha HA, Bellec A, Munhoz CdF, Pedrosa-Harand A, Bergès H, Vieira MLC: **Begin at the beginning: A BAC-end view of the passion fruit (Passiflora) genome**. *BMC Genomics* 2014, **15**(1):816-816.
2. Chen B, Zhou Z, Ke Q, Wu Y, Bai H, Pu F, Xu P: **The sequencing and de novo assembly of the Larimichthys crocea genome using PacBio and Hi-C technologies**. *Sci Data* 2019, **6**(1):188-188.
3. Zhao P, Li J, Kang H, Wang H, Fan Z, Yin Z, Wang J, Zhang Q, Wang Z, Liu J-F: **Structural Variant Detection by Large-scale Sequencing Reveals New Evolutionary Evidence on Breed Divergence between Chinese and European Pigs**. *Scientific Reports* 2016, **6**(1):18501.
4. Amills M, Ramírez O, Galman-Omitogun O, Clop A: **Domestic Pigs in Africa**. *African Archaeological Review* 2013, **30**(1):73-82.
5. Kumar S, Nei M, Dudley J, Tamura K: **MEGA: a biologist-centric software for evolutionary analysis of DNA and protein sequences**. *Brief Bioinform* 2008, **9**(4):299-306.
6. Yang J, Lee SH, Goddard ME, Visscher PM: **GCTA: a tool for genome-wide complex trait analysis**. *Am J Hum Genet* 2011, **88**(1):76-82.
7. Alexander DH, Novembre J, Lange K: **Fast model-based estimation of ancestry in unrelated individuals**. *Genome Res* 2009, **19**(9):1655-1664.
8. Zhao P, Yu Y, Feng W, Du H, Yu J, Kang H, Zheng X, Wang Z, Liu GE, Ernst CW *et al*: **Evidence of evolutionary history and selective sweeps in the genome of Meishan pig reveals its genetic and phenotypic characterization**. *Gigascience* 2018, **7**(5).
9. Zhao Z, Fu YX, Hewett-Emmett D, Boerwinkle E: **Investigating single nucleotide polymorphism (SNP) density in the human genome and its implications for molecular evolution**. *Gene* 2003, **312**:207-213.
